# Progress in Medicine

**Published:** 1895-06-08

**Authors:** 


### Progress in Medicine.

### DISEASES OF CHILDREN.

**Infantile Scurvy.**—In the Bradshaw lecture for 1894 Dr. Thomas Barlow[1] has discussed very fully the subject of infantile scurvy, and paid special attention to the relation of that disease to rickets. The characteristic lesion is hæmorrhage under the periosteum of the long bones, but in some cases the bones may be unaffected, and extravasation of blood may occur in the lungs, the kidneys, the intestines, the cerebral membranes, or the orbit. Difficulties arise in the diagnosis of the "borderland" cases—those in which rachitic symptoms are present, but also an amount of irritability and tenderness in the limbs out of all proportion to what is usually found in rickets. In such cases "the substitution or increase of what may be called 'living food' in the diet will often entirely eliminate this irritability and tenderness." He considers the disease to be dietetic in origin, and amongst the foods which induce scurvy he mentions condensed milk, very dilute cow's milk, peptonised milk, and humanised sterilised milk, when these are employed for too long a period. Mr. Howard Marsh[2] has directed attention to infantile scurvy as it presents itself in surgical practice. He has met with cases erroneously diagnosed as fracture of the femur, infantile paralysis, sarcoma of the femur, sarcoma of the eyeball, sarcoma of the gums, and angular curvature of the spine. He does not regard surgical treatment as necessary or advisable. Dr. Baginsky[3] is of opinion that infantile scurvy is becoming more common in Berlin, and in all his cases he has been able to exclude the possibility of the affection being syphilitic. At the same time, he notes the resemblance between the loss of power in the limbs in scurvy and that present in Parrot's syphilitic pseudo-paralysis. His cases were mostly amongst well-to-do people, and the patients had been fed for a considerable time on artificial foods. A specimen of extensive subdural hæmorrhage from a case of infantile scurvy was shown by Dr. Wallis Ord[4] at the Pathological Society of London. The clot occupied the whole of the cranial vault, but had not caused any symptoms during life, and death occurred from an attack of acute broncho-pneumonia. The patient had been fed from birth on a prepared food, but a twin brother brought up on the same diet was quite free from any signs of scurvy.

The views of Dr. Cheadle and Dr. Barlow on the subject of infantile scurvy have received a great amount of support in this country, and in America and Germany, but recently some articles have appeared in which the identity of the conditions described with those of true scurvy have been disputed. Dr. Henry Ashby[5] is of opinion that "the presence of scurvy in these cases has not been certainly proved; that in some cases at least there has been no deprivation of fresh food, and that there is much to be said in favour of the view that there is a close connection between this hæmorrhagic condition and acute rickets." He finds that similar hæmorrhages occur in infants in connection with other diseases, such as syphilis and tuberculosis. Further, in some of his cases fresh milk, raw meat juice, and potatoes entered into the diet, so that the antiscorbutic element was supplied. As regards the results of treatment, he notes that in some cases improvement is slow, even under antiscorbutic diet, and he is inclined to lay as much stress on fresh air as on fresh orange juice. Dr. L. Conitzer[6] has described two cases of what he calls "Barlow's Disease," but which he does not consider to be scurvy. The patients were aged nine and eleven months respectively, and presented signs of rickets. In one the limbs were swollen and the urine contained blood; in the other extreme tenderness was present, the mucous membrane of the gums was swollen and blue, and swelling existed in the lower limbs. These patients recovered under ordinary tonic treatment, and as the symptoms had arisen in one case while the child was on a diet including potatoes, milk, and soup, the writer sees no reason for diagnosing scurvy. Von Starck[7] has had several cases of rachitic infants presenting the symptoms of "Barlow's Disease" along with those of syphilis. Three such patients recovered completely under mercurial treatment (calomel, gr. one-sixth, thrice daily), without the adoption of any change in the diet. He is not inclined to admit the presence of true scurvy in these cases, but to regard them as examples of acute rickets with a hæmorrhagic diathesis.

**Intestinal Disorders.**—Dr. Soltan Fenwick[8] has drawn attention to the dyspepsia of strumous children, an affection to which little reference is made in the text-books. The patients have usually a marked hereditary tendency to tuberculosis, and suffer from local manifestations of the disease, such as enlarged cervical glands and tonsils, phlyctenular ulcers on the cornea, &c. Acute abdominal pain is one of the most constant symptoms. It comes on suddenly, and may last for a few minutes or for several hours; it is griping or twisting in character, and is situated in the region of the transverse colon, the right iliac fossa, and the hypogastrium. An attack frequently follows the ingestion of food, and an urgent call to relieve the bowels is often present at the same time. Diarrhœa is common, and the rapidity with which food is hurried through the stomach and intestines, owing to abnormal peristaltic action, prevents complete digestion and absorption, and maintains a condition of ill-health. In the treatment Dr. Fenwick lays stress on the importance of hygienic measures and the value of sea air. White fish, tender meat, milk and cocoa, vegetables in moderation, gruel, and simple food generally are allowed; but all indigestible foods, amongst which he includes coffee, strong tea, condiments, and spices are to be forbidden. For the torpid condition of the large intestine he orders the fluid extract of cascara sagrada (ten to fifteen minims) in one or two drachms of extract of malt. When the bowels are moved after each meal he employs nepenthe or chlorodyne, with or without soda and bismuth. After the local troubles have been relieved, the anæmia, which is very persistent, must be treated with the tartrate, carbonate, or ammonio-citrate of iron.

[1] The Lancet, 1894, II., p. 1075. [2] B.M.J., Dec. 1, 1894. [3] Berl. Klin. Woch., Feb. 18, 1895, and Ep. B.M.J., March 9, 1895. [4] The Lancet, 1894, II., p. 1483. [5] The Practitioner, Dec., 1894. [6] Mürch Med. Woch., 1894, Nos. 11 12, and Am. Jour. Med. Sci, Oct., 1894. [7] Jahob. f. Kinderheilk, B. 37, Heft. 1, B. 38, Hefte 2, 3, and Med. Chron., Jan., 1895. [8] Clin. Jour., Oct. 24, 1894.

